# Valproic Acid and Famotidine Drug-Drug Interaction: Report of a Pediatric Case

**DOI:** 10.7759/cureus.101236

**Published:** 2026-01-10

**Authors:** Mirei Kato, Lisa Mannina, Shelease O'Bryant, David Hart, Amber Mirajkar

**Affiliations:** 1 Emergency Medicine, Touro College of Osteopathic Medicine, Las Vegas, USA; 2 Emergency Medicine, Sunrise Health Graduate Medical Education (GME) Consortium, Hospital Corporation of America (HCA) Healthcare, Las Vegas, USA; 3 Pediatric Emergency Medicine, Sunrise Health Graduate Medical Education (GME) Consortium, Hospital Corporation of America (HCA) Healthcare, Las Vegas, USA

**Keywords:** cytochrome p450, drug interaction, emergency medicine, famotidine, l-carnitine, pediatric emergency medicine, pharmacology, toxicology, valproic acid, valproic acid toxicity

## Abstract

Valproic acid is a broad-spectrum anticonvulsant medication that is used to treat multiple neurologic and psychiatric disorders such as epilepsy, bipolar disorder, schizophrenia, and migraine prophylaxis. Famotidine is an antacid used for various gastrointestinal conditions, including gastric and duodenal ulcers and gastroesophageal acid reflux disease (GERD). Acute ingestion of famotidine was found to inhibit anticonvulsant action and increase brain concentrations relative to free plasma levels in a mouse model. While they are not known to interact with one another, one of their proposed metabolisms is through the same CYP450 enzyme. However, a drug-drug interaction between valproic acid and famotidine, or this interaction causing toxicity in a child, is extremely rare.

A 10-year-old male with a past medical history of bipolar disorder, posttraumatic stress disorder, and anxiety presented to a community emergency department (ED) with acute altered mental status and increasing somnolence. Routinely taking valproic acid, he had started prescription famotidine two days prior. In the ED, his valproic acid levels were six times the upper limit of normal with associated elevated creatinine and hypocalcemia. Later, hyperammonemia developed. He was ultimately treated with L-carnitine, lactulose, and meropenem as well as admitted to the pediatric intensive care unit (PICU) with normalization of lab values and complete resolution of his neurologic symptoms after 29 hours.

This case describes an extremely rare drug-to-drug interaction of valproic acid and famotidine leading to acute altered mental status in a pediatric patient. While causality cannot be established from a single case, clinicians should consider this potential interaction when new medications are added to a stable valproic acid regimen, as these two medications are commonly prescribed in both adults and children.

## Introduction

Valproic acid is a first-generation anticonvulsant medication frequently used to treat neurologic and psychiatric disorders. The use of valproic acid was discovered by Meunier and Carraz in 1967 and approved by the Food and Drug Administration (FDA) in 1978 to treat absence seizures [1]. Since then, valproic acid has been used as a first-line treatment and adjunctive therapy for multiple seizure disorders, bipolar disorder, and migraine prophylaxis [2].

Valproic acid has multiple mechanisms of action that are not yet fully understood, including the inhibition of voltage-gated sodium channels, gamma-aminobutyric acid (GABA) transaminase, and histone deacetylase enzymes, as well as the enhancement of GABA synthesis and modulation of calcium channels involved in neuronal signaling [1]. It is eliminated primarily via hepatic glucuronidation (UGT1A6/1A9/2B7) and mitochondrial beta-oxidation, with minor hepatic cytochrome P450 (CYP450), such as CYP1A2, involvement [1,3].

Greater than 10% of those taking valproic acid can experience headache, dizziness, tremor, asthenia, nervousness, nausea, vomiting, diarrhea, constipation, abdominal pain, thrombocytopenia, ecchymosis, alopecia, weight changes, emotional lability, insomnia, depression, and appetite changes. Rarely, in less than 1% of those taking it, valproic acid can cause hepatotoxicity, acute psychosis including hallucinations, anaphylaxis, and encephalopathy thought secondary to hyperammonemia from the hepatotoxicity [1].

A few medications increase valproic acid plasma levels to cause toxicity. Two notable examples are felbamate and aspirin [4,5]. Aspirin, specifically at doses of 11-16 mg/kg, can increase the levels through protein binding and inhibiting valproic acid's metabolism by beta-oxidation. Other proposed medications that can indirectly increase valproic acid plasma levels are carbapenem antibiotics and estrogen-containing hormonal contraceptives. Although the mechanisms are not understood, they are thought to inhibit valproic acid's metabolism in the liver [5].

Famotidine is an antacid medication that has been used for various gastrointestinal conditions such as gastroesophageal acid reflux disease (GERD), gastric ulcers, and duodenal ulcers. Famotidine decreases gastric acid production and secretion by blocking histamine H2 receptors located on the basolateral membrane of the stomach's parietal cells. It can reduce the efficacy of medications that require the low pH of gastric acid for bioavailability, such as dasatinib, delavirdine mesylate, cefditoren, fosamprenavir, atazanavir, erlotinib, ketoconazole, itraconazole, ledipasvir/sofosbuvir, nilotinib, and rilpivirine [5].

Current literature supports that famotidine is metabolized by numerous enzymes in the CYP450 system, including CYP1A2, but usually has minimal inhibitory effects on the metabolism of other drugs because it does not bind to the cytochromes. Nevertheless, through this mechanism, it can increase the blood levels of medications like tizanidine, thus causing an overdose-like presentation of hypotension, bradycardia, and drowsiness [6-8]. However, famotidine has not been reported to interact with antiepileptics. Unlike other H2 receptor blockers, such as cimetidine, that do bind to multiple cytochrome enzymes, famotidine is preferred and is considered a safe antacid agent [6,7]. Famotidine is primarily renally excreted after being metabolized by the liver. Side effects include dizziness, headache, constipation, and diarrhea [5]. 

We report a suspected interaction between famotidine and valproic acid by an unknown mechanism, but possibly a greater inhibitory effect of CYP450 enzymes through competition than previously reported, specifically CYP1A2. Pharmacology databases like Clinical Pharmacology powered by Clinical Key® do not report an interaction between these two medications, and there are no known human cases. However, both famotidine and valproic acid have metabolism mechanisms involving the liver's CYP450 system and are both renally excreted. Only an animal model demonstrated a possible example of this interaction, whereby famotidine 5 mg/kg was found to have elevated valproic acid levels in mice brains even after one day, and valproic acid's anticonvulsant activity was enhanced, as measured by the decrease in mean effective dose (ED_50_) [9]. Our case highlights an unusual presentation and clinical course of valproic acid toxicity, reveals a suspected drug-drug interaction between valproic acid and famotidine, and underscores a multitude of treatments of valproic acid toxicity so as to prevent morbidity and mortality.

## Case presentation

A 10-year-old male with a past medical history of bipolar disorder, posttraumatic stress disorder, attention deficit disorder, and anxiety presented to a community ED via ambulance for acute altered mental status and dizziness one morning. The patient was at his mental baseline the night prior, but upon waking, two hours prior to arrival (PTA), he became increasingly somnolent. The patient was closely followed by a psychiatrist and had been treated with valproic acid for a year with no side effects. Approximately one month PTA, his plasma valproic acid levels were subtherapeutic at 30 mcg/ml (therapeutic range 50-100 mcg/ml). However, the dose was increased from 250 mg twice daily (BID) to 500 mg BID two weeks PTA. Per the patient's mother, who controls his medications, there were no accidental or intentional extra doses of valproic acid; she brought the bottles, and the pill count was found to be appropriate. The only addition to his medication regimen was famotidine 10 mg BID two days PTA for abdominal pain and poor oral intake. His other chronic medications included amantadine 100 mg BID and cyproheptadine 4 mg three times daily (TID).

En route to the ED, emergency medical services (EMS) administered naloxone, but there was no change in the patient's sleepiness. At presentation, the patient only responded to the sternal rub by opening his eyes, saying his name, and intermittently following commands. This resulted in a Glasgow Coma Score (GCS) of 11 (eyes: 2; verbal: 4; motor: 5). Initial vital signs were: heart rate 124 beats/minute, blood pressure 130/80 mmHg, respiratory rate 18 breaths per minute, oxygen saturation 99% on ambient air, and temperature 36.3 °C. There were no signs of physical trauma. Point-of-care glucose was normal at 92 mg/dL.

**Figure 1:**
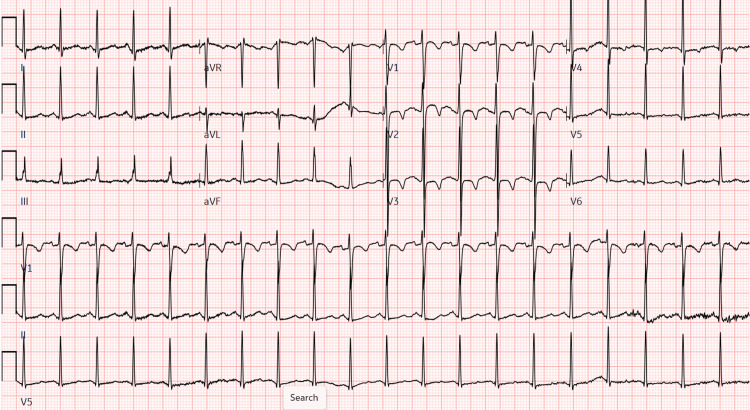
ECG at hour 0 upon community ED arrival Heart rate (HR) 120, PR 122, QRS 72, QTc 452, normal axis.

Initially, laboratory studies (Table 1), an electrocardiogram (ECG, Figure 1), and imaging were obtained. The valproic acid level was elevated at 606.5 µg/ml. Ammonia levels were normal at 23 µmol/L (reference range: 15-45 µmol/L). The complete metabolic panel (CMP) was significant for an elevated creatinine of 0.92 mg/dL (reference range: 0.32-0.64 mg/dL) and hypocalcemia of 7.9 mg/dl (reference range: 9.0-10.1 mg/dL). For comparison, one month PTA, calcium levels were normal at 9.1 mg/dl. The urine toxicology screen was negative, including opiates (usually non-synthetic), barbiturates, phencyclidine, amphetamines, benzodiazepines, cocaine, and cannabinoids. Blood levels of aspirin, acetaminophen, and alcohol were also negative. ECG showed sinus tachycardia with a heart rate of 120, otherwise normal for a pediatric patient (Figure 1). Non-contrast head computerized tomography (CT) showed no acute intracranial pathology. With the elevated valproic acid level, valproic acid toxicity was the leading diagnosis. At Poison Control's recommendation, the patient was given L-carnitine, the antidote for valproic acid, at a 100 mg/kg intravenous (IV) loading dose, then 50mg/kg IV every eight hours until clinical and laboratory improvement. At the community ED, two doses were administered: 3000 mg IV bolus then 1500 mg IV, calculated for his weight of 40.4kg. The patient's cardiopulmonary and neurologic statuses were monitored, and the patient was transferred to a tertiary pediatric children's hospital.

**Table 1:** Trended laboratory test results throughout the visit PICU - pediatric intensive care unit; CMP - complete metabolic panel; BUN - blood urea nitrogen; AST - aspartate aminotransferase; ALT - alanine aminotransferase; CBC - complete blood count; WBC - white blood cells; H - higher than the reference range; L - lower than the reference range

Laboratory test	Arrival at community ED (hour 0)	Arrival at tertiary hospital (hour 9)	PICU (hour 14)	PICU (hour 18)	Normal range
CMP					
Sodium	138 mmol/L	142 mmol/L	142 mmol/L		136-145 mmol/L
Potassium	4.0 mmol/L	3.7 mmol/L	3.5 mmol/L		3.5-5.5 mmol/L
Chloride	105 mmol/L	112 mmol/L (H)	113 mmol/L (H)		93-107 mmol/L
Carbon dioxide	24 mmol/L	23 mmol/L	24 mmol/L		21-32 mmol/L
Anion gap	13 mmol/L	7 mmol/L	5 mmol/L		5-16 mmol/L
BUN	8 mg/dL	6 mg/dL	5 mg/dL		6-17 mg/dL
Creatinine	0.92 (H) mg/dL	0.75 mg/dL	0.58 mg/dL		0.2-0.75 mg/dL
Glucose	92 mg/dL	84 mg/dL	100 mg/dL		60-100 mg/dL
Calcium	7.9 mg/dL (L)	8.9 mg/dL (L)	7.4 mg/dL (L)		9.0-10.1 mg/dL
Total bilirubin	0.2 mg/dL	0.2 mg/dL			0.1-1.2 mg/dL
AST	25	20			10-36 U/L
ALT	16	19			16-61 U/L
Total ALK phosphatase	281	237			98-317 U/L
Lipase	54				13-75 U/L
CBC					
WBC	5.8x10^3^/µL	5.8x10^3^/µL			4.27-13.5 x10^3^/µL
Hemoglobin	15.2 gm/dL	13.3 gm/dL			11.5-14.5 gm/dL
Platelets	238x10^3^/µL	223x10^3^/µL			150-450 x10^3^/µL
Toxicology					
Ammonia	23 µmol/L	15 µmol/L	157 µmol/L (H)	81 µmol/L (H)	11-32 µmol/L
Valproic acid	606.5 µg/mL (H)	258 µg/mL (H)	175 µg/mL (H)	109 µg/mL (H)	50-100 µg/mL

At hour nine after initial presentation to the community ED, the patient arrived at the pediatric ED of the tertiary hospital. The repeat valproic acid level was 258 µg/mL, ammonia levels remained normal at 15 µmol/L, and creatinine normalized to 0.75 mg/dl. Repeat ECG was unchanged. By hour ten, he had returned to his mental status baseline with a normal GCS of 15 (eyes: 4; verbal: 5; motor: 6). At that time, Poison Control's recommendations were to observe the patient, correct electrolyte abnormalities, and monitor ammonia levels until valproic acid levels normalized.

**Figure 2:**
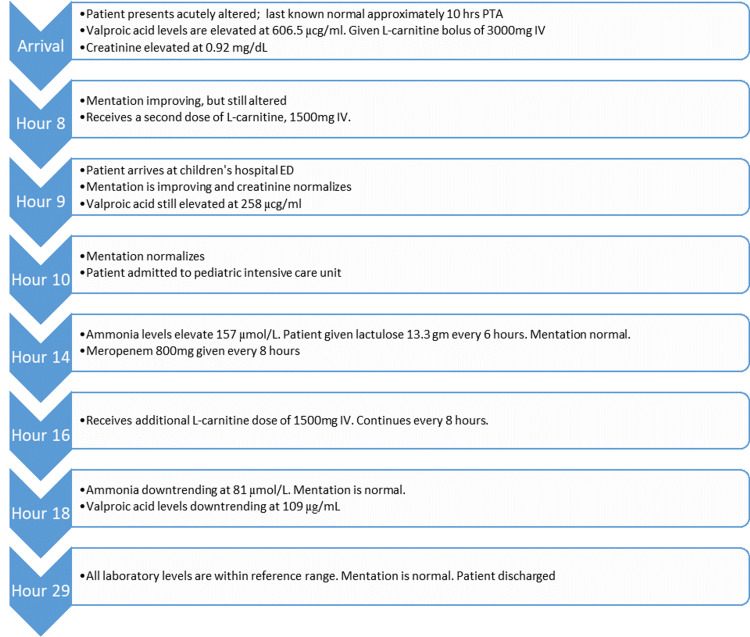
Emergency department and hospitalization timeline

The patient was admitted to the pediatric intensive care unit. During hospitalization (Figure 2), at hour fourteen, ammonia levels became elevated to 157 µmol/L; thus, oral lactulose 13.3 g for hyperammonemia every six hours and 800 mg meropenem IV every eight hours for valproic acid toxicity were administered. After treatment, valproic acid and ammonia levels downtrended, with improvement and normalization to 29 µg/ml and 39 µmol/L, respectively, at hour twenty-nine. The patient was placed on an electrolyte regimen, and calcium levels normalized. There were no further complications during the patient's admission. The patient's psychiatrist recommended discontinuing the valproic acid, and the patient was discharged home with primary care and psychiatric follow-up on the second hospital day.

This patient was evaluated in the same hospital system approximately nine months later, with no further reported toxicological symptoms, although no laboratory testing was performed.

## Discussion

While valproic acid is generally well tolerated, valproic acid toxicity can be difficult to diagnose with its numerous side effects ranging from mild to life-threatening (Table 2). Thrombocytopenia shows the clearest plasma concentration-dependent relationship, with the probability increasing at valproic acid concentrations greater than 110 µg/ml in females or greater than 135 µg/ml in males. Hepatotoxicity and hyperammonemia have weaker correlations with plasma levels, but nevertheless, monitoring plasma levels is only useful in these three situations [4]. Our patient did not have thrombocytopenia or hepatotoxicity, but his hyperammonemia was trended along with his plasma valproic acid concentration. Otherwise, the blood plasma levels do not correspond with other side effects or their severity level.

**Table 2:** Adverse effects of valproic acid Source: [1]

System	Effects
General	Weight changes, electrolyte abnormalities, appetite changes, hypothermia, fever
Respiratory	Respiratory depression, flu syndrome, infection, bronchitis, rhinitis
Gastrointestinal	Nausea, vomiting, diarrhea, abdominal pain, pancreatitis, hepatotoxicity (rare), transaminitis, anorexia, dyspepsia, constipation
Dermatologic	Rash, petechiae, ecchymosis, Stevens-Johnson syndrome, erythema multiforme, alopecia
Neurologic	Ataxia, headache, tremors, sedation, photosensitivity, tinnitus, diplopia, blurred vision, nystagmus, amnesia, emotional lability, cerebral edema, encephalopathy, coma
Hematologic	Agranulocytosis, thrombocytopenia, myelosuppression, aplastic anemia, bleeding
Psychologic	Depression, hallucinations
Reproductive	Polycystic ovarian syndrome, teratogenicity

Valproic acid, and by extension, anti-epileptics, are not included in the top five substance categories most commonly involved in toxicities (i.e. analgesics, personal care products, cleaning substances, sedatives/hypnotics, and foreign bodies) according to the 2010 Annual Reports of the American Association of Poison Control Centers' National Poison Data System 28th Annual Report and thus may not immediately be considered by clinicians [10]. In 2010, there were approximately 3,211 cases of acute valproic acid ingestion; this was 0.13% of all reported ingestions [2,10]. Although valproic acid toxicity is uncommon, it is important to recognize an overdose and identify any drug interactions due to its lethal effects.

Our case illustrates a proposed drug-drug interaction between valproic acid and famotidine that has not been previously reported in humans. One of the most common metabolism mechanisms in the body is through the liver's CYP450 system. Famotidine is metabolized by the CYP450 system, including CYP1A2, and then renally excreted [5-7]. Valproic acid is metabolized almost entirely in the liver through multiple pathways involving glucuronidation via the enzyme UDP-glucuronosyltransferase (UGT), mitochondrial beta-oxidation, and conjugation with carnitine [1, 2]. Once the liver processes valproic acid, its metabolites are excreted in the urine. However, valproic acid is a weak inhibitor of various cytochrome CYP450 enzymes, including but not limited to CYP1A2, CYP2C9, and CYP2A6 [1,3-5]. It is possible that our suspected interaction between these two medications, leading to valproic acid toxicity, is in the CYP450 system.

In this case, the patient was compliant with his medication regimen and closely followed by his psychiatrist. While the patient's dose of valproic acid had been doubled from 250 mg BID to 500 mg BID two weeks PTA, it is unlikely to have caused the toxicity alone. Steady state of a medication is generally achieved after four to five half-lives; the half-life for valproic acid is nine to sixteen hours for his dose. Thus, steady state would have been reached within 1.5 to 3.5 days, as opposed to fourteen days later [5]. Furthermore, a month prior to arrival, the patient's valproic acid levels were subtherapeutic at 30 µg/ml. The mental status changes associated with valproic acid-induced hyperammononemic encephalopathy occur insidiously, as opposed to the acute change seen in our patient [5]. The only identified immediate change in his medication regimen around the time of the patient's mental status change was the addition of famotidine for two days prior to his hospitalization.

While the patient was also taking amantadine 100 mg BID and cyproheptadine 4 mg TID, there are no established interactions with these medications and valproic acid according to Clinical Pharmacology powered by Clinical Key®. Amantadine is a non-competitive antagonist of the N-methyl-D-aspartate receptor (NMDA), leading to dopamine release, and is metabolized by the kidney [11]. This mechanism is very different than that of valproic acid and famotidine. Cyproheptadine has anticholinergic, anti-serotonergic, and anti-histamine properties. While it is mainly metabolized in the liver via glucuronidation, it rarely causes hepatic toxicity, and if it does, it usually occurs in the first six weeks of administration [12]. Furthermore, the patient had been taking valproic acid, amantadine, and cyproheptadine for approximately one year without side effects.

In valproic acid toxicity, the most common manifestation is central nervous system (CNS) dysfunction, ranging from mild drowsiness to cerebral edema and death [1]. CNS depression is often seen when there is an acute ingestion of at least 200 mg/kg or a valproic acid level greater than 180 µg/ml [2]. Our patient's first level was 606 µg, so his sleepiness and altered state could have been from the valproic acid accumulation alone. Given the close control of the medication by his mother, there were concerns for altered metabolism causing phenocopy, or the semblance of an overdose without an actual overdose.

As in our patient, valproic acid-associated hyperammonemia may also occur. Hyperammonemia occurs from valproic acid inhibiting N-acetylglutamate synthetase (NAGS), the enzyme necessary for the clearance of ammonia through the urea cycle using carnitine. Without NAGS, ammonia can accumulate and lead to encephalopathy [13-15]. However, asymptomatic hyperammonemia has also been seen in those taking valproic acid chronically, and the temporal-dose relationship is not well established [1]. Ultimately, the two levels declined concurrently starting on the eighteenth hour. The patient's mental status had returned to normal baseline at hour ten, and the hyperammonemia occurred at hour fourteen, so the delayed hyperammonemia is unlikely to have caused the increasing somnolence. Why there was a cunctation is unclear. It is also possible that the hyperammonemia was delayed because the L-carnitine given as an antidote provided the carnitine needed, as those chronically on valproic acid have their endogenous carnitine stores depleted [15]. While this was not the typical liver failure-induced hyperammonemia, lactulose was still given with improvement of ammonia levels, as lactulose acidifies the gut to decrease ammonia production in addition to trapping ammonia in the intestine's lumen. 

According to Świąder et al., acute ingestion of famotidine (5 mg/kg) significantly elevated the brain concentration of valproate without effects on its free plasma levels in mice after just one day [9]; however, this is not routinely tested in humans. While it can be difficult to extrapolate from mouse models, this interaction may explain the altered mental state of the patient, even before ammonia levels were elevated. As none of the other medications our patient was taking had an interaction with valproic acid, famotidine was added to the patient's medication regimen days before his presentation, and there is an animal model demonstrating an interaction between famotidine and valproic acid, it was our concern that this interaction caused the patient's condition. Furthermore, in the twelve hours to four days after a critical ingestion of famotidine, a CT head may show cerebral edema [7,10]. However, the CT head on this patient did not show any pathology, possibly indicating that a properly dosed amount of famotidine could still cause an altered mental status.

The treatment for valproic acid toxicity should start with assessing and optimizing cardiopulmonary and neurologic status. Naloxone, although not the mainstay treatment, can help reduce CNS depression, but was ineffective in our patient [16]. Another option is L-carnitine, an essential cofactor in the beta-oxidation of fatty acids that valproic acid inhibits. It is proposed that L-carnitine supplementation in acute valproic acid toxicity can increase the beta-oxidation of valproic acid in the liver and limit the production of toxic metabolites, and thus prevent the development of hepatic dysfunction [17,18]. One regimen for pediatric patients without hepatotoxicity is 100 mg/kg divided by four to be administered every six hours IV until there are declining laboratory values and clinical improvement, with clinical improvement typically seen within 12 hours. For those with hyperammonemia and/or hepatotoxicity, one regimen is a loading dose of six grams and then 15 mg/kg every four to six hours IV until there is lab and clinical improvement. Another regimen in this cohort is 50 mg/kg/dose (maximum of three grams per dose) IV every eight hours until lab and clinical improvement, but it is unclear which is more effective [19]. It is possible our patient achieved mental status improvement quickly because he had a loading dose of 100 mg/kg IV, then 50 mg/kg IV every eight hours, a combination of multiple of the aforementioned regimens.

If valproic acid levels exceed 1300 µg/mL, the patient is in shock, or the CT head shows cerebral edema, then hemodialysis is indicated [2]. It can accelerate improvement by hours and prevent further organ damage. Hemodialysis is no longer needed once valproic acid levels decrease below 100 µg/mL, electrolytes normalize, vital signs normalize, and/or mentation improves [2]. Luckily, our patient did not need hemodialysis as an antibiotic was tried instead.

There have been new studies showing that meropenem may have a role in valproic acid toxicity [20]. Carbapenems can decrease the efficacy of valproic acid clinically; it is thought that meropenem inhibits acylpeptide hydrolase in the liver, increasing liver enterohepatic recirculation, which then can lower valproic acid concentrations as much as 50-80% [20-22]. Amongst the carbapenems, meropenem and ertapenem are not only more efficacious in this process but also less epileptogenic than imipenem. However, regardless of whether meropenem or ertapenem is chosen, increasing doses do not lead to faster or improved clearance, so typically one gram every eight hours is administered [21]. As in our patient, meropenem was used to accelerate valproic acid clearance after the development of hyperammonemia, with subsequent success in laboratory values. Given the delay in administration in both our case and the literature, meropenem is unlikely to reduce absorption. The few studies that show the benefits of meropenem in valproic acid toxicity added the antibiotic because, despite conventional therapy, the valproic acid plasma levels continued to increase, the mental status continued to decline, or both [20,21]. As it is an off-label use for meropenem, the general dose of 20 mg/kg is recommended, but use should be individualized and balanced with antimicrobial stewardship principles. A combination of L-carnitine and meropenem was able to stave off dialysis in this pediatric patient. Altogether, advances in treatments have expanded options for managing overdose and non-overdose valproic acid toxicity as well as reducing associated morbidity and mortality.

## Conclusions

The current case highlights a possible temporal drug-drug interaction between famotidine and valproic acid, leading to valproic acid toxicity and acute encephalopathy in a pediatric patient chronically taking valproic acid. Although the mechanism is unknown and there are confounding factors, this case emphasizes the risks associated with altering medication regimens that include valproic acid treatment, especially as it is commonly prescribed to patients of all ages. Further pharmacovigilance and mechanistic studies are needed; however, until then, administration of L-carnitine, lactulose, and meropenem may be helpful in the treatment of acute valproic acid toxicity, even in a non-overdose situation, and its corresponding hyperammonemia.
